# Site-Specific Chemoenzymatic Labeling of Aerolysin Enables the Identification of New Aerolysin Receptors

**DOI:** 10.1371/journal.pone.0109883

**Published:** 2014-10-02

**Authors:** Irene Wuethrich, Janneke G. C. Peeters, Annet E. M. Blom, Christopher S. Theile, Zeyang Li, Eric Spooner, Hidde L. Ploegh, Carla P. Guimaraes

**Affiliations:** Whitehead Institute for Biomedical Research, Department of Biology, Massachusetts Institute of Technology, Cambridge, Massachusetts, United States of America; Institut Curie, France

## Abstract

Aerolysin is a secreted bacterial toxin that perforates the plasma membrane of a target cell with lethal consequences. Previously explored native and epitope-tagged forms of the toxin do not allow site-specific modification of the mature toxin with a probe of choice. We explore sortase-mediated transpeptidation reactions (sortagging) to install fluorophores and biotin at three distinct sites in aerolysin, without impairing binding of the toxin to the cell membrane and with minimal impact on toxicity. Using a version of aerolysin labeled with different fluorophores at two distinct sites we followed the fate of the C-terminal peptide independently from the N-terminal part of the toxin, and show its loss in the course of intoxication. Making use of the biotinylated version of aerolysin, we identify mesothelin, urokinase plasminogen activator surface receptor (uPAR, CD87), glypican-1, and CD59 glycoprotein as aerolysin receptors, all predicted or known to be modified with a glycosylphosphatidylinositol anchor. The sortase-mediated reactions reported here can be readily extended to other pore forming proteins.

## Introduction

Pore-forming toxins (PFTs) comprise the largest category of bacterial virulence factors [1]. One of the better studied examples is aerolysin secreted by *Aeromonas hydrophila* [2]. Aerolysin forms a homo-heptameric pore that spans the plasma membrane of the target cell [3] [4], leading to depletion of small ions [5] [6] [7], rapid loss of ATP, and ultimately cell death [8].

**Figure 1:**
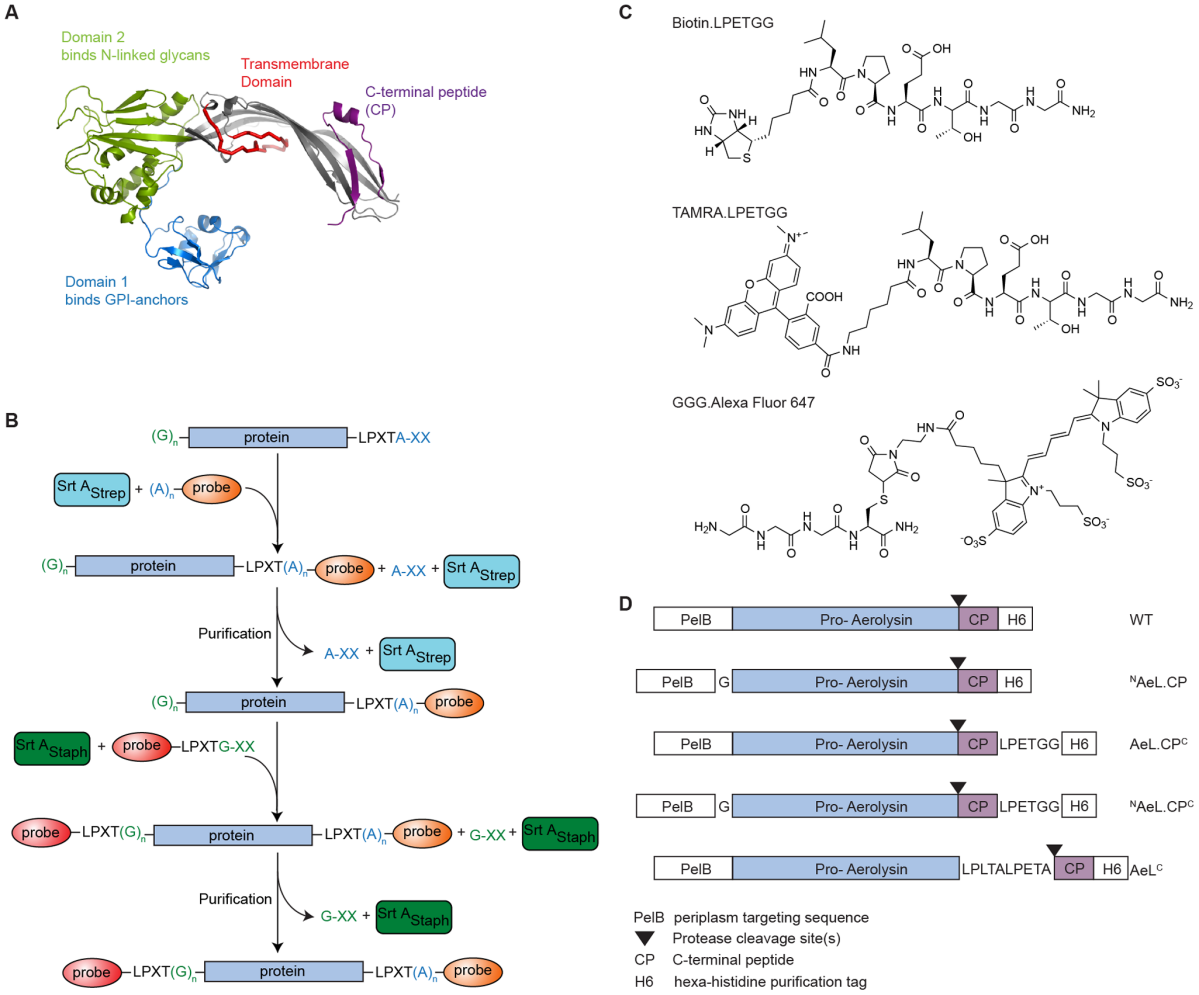
Strategies for site-specific labeling of proaerolysin. **A** Structure of the proaerolysin monomer (PDB: 1PRE). Proaerolysin consists of several different domains, two of which are responsible for receptor binding (domains 1 and 2), one containsing the trans-membrane domain, and the C-terminal peptide (CP), which functions as a chaperone and dissociates from the rest of the complex upon heptamer association and pore formation. **B** Sortase reaction mechanism. C-terminal sortagging: sortase cleaves after threonine in the context of its recognition motif resulting in the formation of a new covalent bond with the N-terminus of an added oligoglycine or oligoalanine nucleophile coupled to a label of choice. N-terminal sortagging: the N-terminal glycine of proaerolysin is recognized as a nucleophile by sortase and conjugated to an LPXTG/A probe bearing a label. **C** Structures of probes used in this study. Not depicted is AAA.Alexa Fluor 647, which is similar to GGG.Alexa Fluor 647, but with alanine replacing glycine. PelB: periplasm targeting sequence, cleaved off by the producer bacteria upon export of proaerolysin to the periplasm. H6: hexahistidine handle for affinity purification. Protease cleavage sites are recognized by target cell surface proteases such as furin. CP: C-terminal peptide, serves as a chaperone for proaerolysin. Upon its loss, proaerolysin is converted to mature aerolysin (AeL). **D** Scheme for wild type (WT) and sortaggable versions of proaerolysin with their designations. The LPXTG/A pentapeptides are sortase recognition motifs.

Aerolysin is secreted as an inactive monomeric precursor, proaerolysin, comprising a 43-residue C-terminal peptide (CP) [9] (Fig. 1*A*). The CP has chaperone features and appears to be required in the course of synthesis to properly fold proaerolysin into its soluble form. It not only prevents aggregation but also impedes premature pore formation by controlling the onset of heptamerization [10]. Proaerolysin is known to bind to N-glycosylated glycosylphosphatidylinositol (GPI)-anchored proteins at the target cell surface [11] [12]. Not only is the glycan important for binding but also the polypeptide to which it is attached [13].

Maturation of proaerolysin to aerolysin involves proteolytic cleavage in a flexible loop that precedes the C-terminal peptide. Furin is thought to play a major role in this process, but other proteases at the plasma membrane may participate as well [14] [15]. Following cleavage, monomers oligomerize to form a prepore complex on the cell surface [16], a step that requires release of the C-terminal peptide [17]. Removal of the C-terminal peptide induces the transition from prepore to the pore complex. The aerolysin heptamer undergoes a drastic concerted conformational change of the extramembranous region, accompanied by a vertical collapse of the complex, which ultimately leads to the insertion of a water-filled transmembrane beta-barrel into the lipid bilayer [17]. The CP is not part of the functional pore, as inferred from tryptophan fluorescence and energy transfer measurements [18]. Its fate after separation from the heptamer is unknown.

Insights into the mechanism of aerolysin intoxication have been obtained without the possibility of labeling discrete domains of the toxin at will. Being able to do so might allow a more detailed examination of the role and fate of each of the specific domains. It is still unclear which domains of aerolysin bind to the proteinaceous moieties of its receptors. Chemical labeling of exposed Lys or Cys residues usually results in a heterogeneous population of labeled proteins, making it impossible to accurately assess the identity of the molecular species responsible for activity. To overcome this technical challenge, we explore sortase-based site-specific chemoenzymatic labeling [19]–[21]. This allows us to investigate the fate of individual N- and C-terminal domains, while preserving toxin activity. Attachment of a single fluorophore at the very C-terminus of the C-terminal peptide makes it possible to directly visualize this chaperone’s departure during aerolysin intoxication. Attachment of a single biotin group at the N-terminus of aerolysin enables us to identify novel cell surface receptors.

## Materials and Methods

### Antibodies, cell lines, constructs

Antibodies against CD59 (sc-28805) and mesothelin (sc-50427) were purchased from Santa Cruz Biotechnology. HRP-coupled secondary anti-rabbit antibody was from BD Biosciences. HeLa cells were purchased from American Type Culture Collection and cultured in Dulbecco’s Modified Eagle Medium (DMEM) supplemented with 10% Fetal Bovine Serum (FBS). KBM7 cells were a kind gift from the T. R. Brummelkamp lab, and were described previously [22]. KBM7 cells were maintained in Iscove’s Modified Dulbecco’s Medium (IMDM) supplemented with 10% FBS. The wild type proaerolysin construct [23] was a generous gift from F. G. van der Goot. Sortaggable variants were cloned by site-directed mutagenesis using the QuikChange kit (Agilent Technologies) following the manufacturer’s instructions and using the following primers: ^N^AeL.CP (introduction of a single glycine at N-terminus),

forward: 5′-AGCCGGCGATGGCCGGTATGGCAGAGCCCGTC-3′,

reverse: 5′-GACGGGCTCTGCCATACCGGCCATCGCCGGCT-3′; AeL.CP^C^ (introduction of LPETGG at C-terminus),

forward: 5′-GCGTGACCCCTGCTGCCAATCAACTACCAGAGACCGGTGGACTCGAGCACCACCACCACCACCACTGAGATCC-3′,

reverse: 5′-GGATCTCAGTGGTGGTGGTGGTGGTGCTCGAGTCCACCGGTCTCTGGTAGTTGATTGGCGCAGGGGTCACGC-3′. ^N^AeL.CP^C^, was built using the forward primer 5′-AGCCGGCGATGGCCGGTATGGCAGAGCCCGTC-3′,

and the reverse primer 3′-GGATCTCAGTGGTGGTGGTGGTGGTGCTCGAGTCCACCGGTCTCTGGTAGTTGATTGGCAGCAGGGGTCACGC-5′ with a PCR on WT proaerolysin template using the Expand High Fidelity PCR system (Roche Diagnostics). AeL.^C^ (introduction of LPLTALPETA motive upstream of the C-terminal peptide) was done in a two-step-manner using QuikChange, according to the manufacturer’s instructions:

5′-AGATCGGTGCTCCCCTCCCGCTCACTGCTGACAGCAAGGGTG-3′,

3′-CACCTTGCTGTCAGCAGTGAGCGGGAGGGGAGCACCGATCT-5′;

5′-TCCCCTCCCGCTCACTGCTCTCCCGGAGACTGCTGACAGCAAGGTGCGTCG-3′,

3′-CGACGCACCTTGCTGTCAGCAGTCTCCGGGAGAGCAGTGAGCGGGAGGGGA-5′.

### Expression and purification proaerolysin

Overnight cultures of *E. coli* BL21 (DE3) pLysS (Promega) transformed with the various aerolysin constructs and grown at 30°C were diluted 1∶50 with LB broth supplemented with 200 µg/mL ampicillin plus 35 µg/mL chloramphenicol, and incubated at 37°C, shaking at 220 rpm, to an optical density of 0.5–0.6 at 600 nm. Expression of proaerolysin was induced with 1 mM isopropyl-beta-D-1-thiogalactopyranoside (IPTG) (Sigma), and the temperature was lowered to 26°C. After 4–5 hours, cells were harvested and centrifuged at 6000×g, 4°C for 20 min. Subsequent steps were carried out at 4°C. Cell pellets were resuspended in 10 ml lysis buffer per 1 L expression culture: 50 mM Tris-HCl pH 7.5, 300 mM NaCl, 0.5 mg/ml polymixin B (Sigma) supplemented with complete protease cocktail inhibitors (Roche) and 50 µg/ml phenylmethylsulfonyl fluoride (PMSF) (Sigma). The suspension was agitated for 45 minutes at 4°C and centrifuged at 6000×g for 30 min at 4°C. The supernatant was incubated at 4°C with 0.25 ml bed volume NiNTA agarose (Qiagen) per 1 L culture, overnight, with gentle rotation. The resin was washed with 20 column volumes of 50 mM Tris-HCl pH 7.5, 300 mM NaCl, 10 mM imidazole. The protein was eluted with 5 column volumes 50 mM Tris-HCl pH 7.5, 300 mM NaCl, 150 mM imidazole. The fractions were subjected to buffer exchange to 50 mM Tris-HCl pH 7.5, 300 mM NaCl, using a PD-10 desalting column (GE Healthcare). 10% (v/v) glycerol was added to the protein preparations, aliquots were snap-frozen, and stored at −80°C. Protein concentration was determined by Bradford assay (Bio-Rad Laboratories).

### Toxicity assay

0.5×10^5^ KBM7 WT cells were incubated for 1 h at 37°C with different concentrations of each of the aerolysin variants (as indicated in the figures) in a total volume of 100 µL. Cells were washed twice with cold PBS and resuspended in PBS containing 1 µg/mL propidium iodide and analyzed by flow cytometry. The percentage of PI negative controls was set to 100%, and the 50% lethal dosis (LC50) calculated in R. 0.001 was added to all concentration values to avoid taking a log2 of 0.

### Flow cytometry

Data acquisition was performed on a FACS Calibur HTS (BD Biosciences) using the CellQuest Pro (BD Biosciences) software. Data were analyzed with FlowJo (Tree Star Inc.).

### Sortase expression, purification, immobilization. Sortase expression, purification, immobilization

Sortase A (SortA) from *Staphylococcus aureus* (SrtA_Staph_) and SortA from *Streptococcus pyogenes* (SrtA_Strep_) were expressed and purified as described previously [21] [20]. Additionally we used a heptamutant form of Sortase A from *S. aureus* (SrtA_staph7M_), which combined previously described mutations to give Ca^2+^ independence and increased activity [24] [25]. SrtA was immobilized on cyanogen bromide activated sepharose beads (Sigma) in a ratio of 1 g dry beads per 30 mg SrtA_Staph_ or 40 mg SrtA_Staph7M_. The beads were swelled in 50 mL of 1 mM HCl for five washes of five minutes each at 4°C. After extensive washing with ice-cold water the sortase was coupled to the beads in 100 mM NaHCO_3_ and 500 mM NaCl for 2 hrs at 25°C or O.N. at 4°C (make sure the storage buffer of the SortA is exchanged as Tris will react with the beads). Finally, the coupled beads were washed and stored as a 50% bead slurry in 50 mM Tris (pH 7.4) and 150 mM NaCl at 4°C. All washes/filtrations were done in a plastic capped fritted column and the buffers were removed between steps by vacuum filtration. For long-term storage more than one week add 20% glycerol and store aliquots at −20°C.

### Synthesis of sortase probes and sortase labeling

GGG.TAMRA, AAA.AF647, TAMRA.LPETGG and Biotin.LPETGG were synthesized as described in [20] [21]. Soluble sortase labeling reactions with SrtA_strep_ and SrtA_staph_ were performed as described [19] [20] [21] [26]. The SrtA_staph7M_ has increased activity and reactions took place at 4°C and with 20% of sortase in relation to proaerolysin. Additionally, Ca^2+^ is no longer needed in the coupling buffer. Sortase immobilized to cyanogen bromide beads was filtered from the reaction solution. Otherwise reaction conditions are the same as the soluble sortase.

### Fluorescence image scan

Fluorescence scans were obtained using a variable mode imager (Typhoon 9200; GE Healthcare).

### SDS PAGE, Coomassie staining, and Immunoblot

SDS-PAGE was performed as described [27]. Gels were stained with Coomassie Brilliant Blue R250 (Thermo Scientific) according to the manufacturer’s instructions. Proteins were blotted onto polyvinylidene difluoride (PVDF) membranes and probed with the appropriate antibodies, followed by chemoluminescence detection using Western Lightning ECL detection kit (Perkin Elmer Life Sciences) and exposure to XAR-5 films (Kodak).

### Fluorescence microscopy

HeLa cells grown on coverslips were washed with ice-cold DMEM media and incubated on ice for 30 minutes with the appropriate concentrations of labeled or unlabeled aerolysin (as indicated in the figures). Cells were washed 3 times with ice-cold PBS, fixed with 4% paraformaldehyde in PBS for 20 minutes at room temperature to prevent activity of plasma membrane-associated proteases that cleave off the C-terminal peptide, washed with PBS, incubated for 1 minute in PBS containing 1 µg/mL Hoechst stain, and mounted with glycerol on coverslips. Alternatively, cells were shifted to 37°C after Hoechst staining. All images were collected on a PerkinElmer Ultraview Multispectral Spinning Disk Confocal Microscope equipped with a Yokogawa CSU-22 spinning disk confocal on a Zeiss Axiovert 200 motorized inverted microscope with Chroma 488/568/647 and 458/515/647 triple dichroic mirrors and Prior emission filter wheel, Perkin Elmer laser launch with 100 mW argon gas laser (488 nm, 514 nm), 100 mW krypton gas laser (568 nm), and 405 nm, 440 nm and 640 nm solid state lasers with AOTF for laser line selection/attenuation and fiber-optic delivery system, a Zeiss 1.4 NA oil immersion 63x objective lens and a Prior piezo-electric objective focusing device for maintenance of focus. Images were acquired with a Hamamatsu ORCA ER cooled CCD camera controlled with Volocity software. Confocal images were collected using an exposure time of 500 ms and 1×1 binning. For time-lapse microscopy, laser power was set to 77% for the 100 mW krypton gas laser (568 nm), and to 100% for the 640 nm 40 mW solid-state laser. Number of frames: 1 per image. Acquisition frequency: 1 frame per 25 seconds. Brightness was adjusted on displayed images (identically for compared image sets) using Fiji software.

### Immunoprecipitation

∼ 10^7^ HeLa cells per condition were incubated with 120 µg ^Biotin^AeL.CP or WT aerolysin for 30 min at 4°C, washed, scraped, and lysed in buffer containing 0.5% (v/v) NP40, 10 mM Tris-HCl pH 7.4, 150 mM NaCl, 5 mM MgCl_2_, supplemented with complete protease cocktail inhibitors (Roche) and 50 µg/ml phenylmethylsulfonyl fluoride (PMSF) (Sigma). Immunoprecipitations were performed for 3 h at 4°C with rotation using 20 µL neutravidin-sepharose beads (Thermo Scientific) per sample. Samples were eluted by boiling in reducing sample buffer and subjected to SDS-PAGE, followed by immunoblotting or mass spectrometry.

### Mass spectrometry

Bands were excised, reduced, alkylated and digested with trypsin at 37C overnight. The resulting peptides were extracted, concentrated and injected onto a Dionex RSLCnano HPLC equipped with a self-packed Jupiter 3 µm C18 analytical column (0.075 mm by 10 cm, Phenomenex). Peptides were eluted using standard reverse-phase gradients. The effluent from the column was analyzed using a Thermo Orbitrap Elite mass spectrometer (nanospray configuration) operated in a data dependent manner. The resulting fragmentation spectra were correlated against the known database using SEQUEST. Scaffold Q+S (Proteome Software) was used to provide consensus reports for the identified proteins.

## Results

### Strategies for site-specific labeling of proaerolysin

Sortases A (SrtA) recognize a pentapeptide motif specific to an individual bacterial enzyme, e.g., LPXTG for SrtA from *Staphylococcus aureus* (SrtA_Staph_) and LPXTA for SrtA from *Streptococcus pyogenes* (SrtA_Strep_) (where X is any aminoacid). SrtA cleaves the peptide bond between the threonine and glycine or alanine, respectively, yielding a thioacyl intermediate, which is then resolved by a nucleophilic attack of the N-terminus of an oligoglycine- or oligoalanine-containing nucleophile (Fig. 1*B*). This results in the formation of a new peptide bond [28] [26]. Because SrtA_Staph_ and SrtA_Strep_ enzymes are orthogonal to one another it is possible to introduce two distinct labels into one and the same protein or virus [29] [30] [31].

Hexa-histidine tags have been genetically installed at the C-terminus of proaerolysin [23]. However, site-specific fluorescent labeling of the C-terminus of mature aerolysin has not previously been attempted, an essential requirement for live-cell imaging. Using sortases we installed biotin and fluorophore probes onto different domains of proaerolysin (Fig. 1*C*). Labels were placed at: the N-terminus of proaerolysin (^N^AeL.CP), the C-terminus of the C-terminal peptide (AeL.CP^C^), the C-terminus of aerolysin preceeding the chaperone (AeL^C^), as well as creating a double-label variant (^N^AeL.CP^C^). The different sortaggable proaerolysin versions are schematically diagrammed in Fig. 1*D*.

### Aerolysin activity

**Figure 2:**
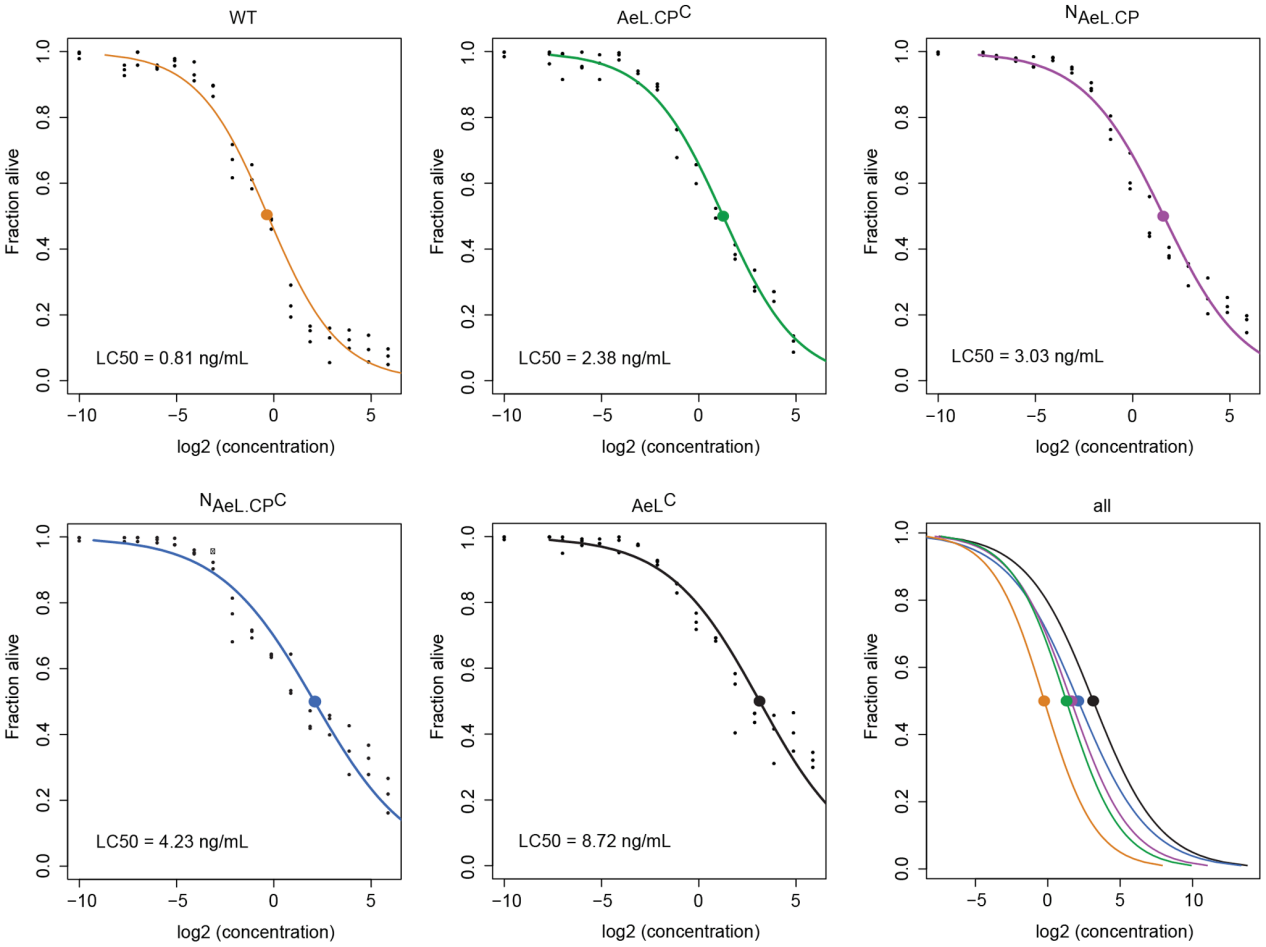
Impact of aerolysin modification on toxic activity. Aerolysin variants were titrated on KBM7 cells. 0.5×10^5^ cells per sample were incubated with toxin for 1 hour at 37°C in a total volume of 100 µL, stained with propidium iodide (PI), and the PI negative percentage determined by flow cytometry. The concentration range for the aerolysin variants ranged from 60 ng/mL to 4 pg/µL. Every condition was tested in triplicate. The percentage of PI negative controls was set to 100%, and the 50% lethal dose (LC50) calculated in R. 0.001 was added to all concentration values to avoid taking a log2 of 0.

The different versions of sortaggable aerolysin were titrated on KBM7 cells. Toxin concentrations ranging from 60 ng/mL to 4 pg/mL were assayed in triplicate. The assay was performed for all aerolysin versions and concentrations in a single experiment on aliquots of the same batch of cells. Cells (3.5×10^5^ per sample) were intoxicated for 1 hour at 37°C, washed, stained with propidium iodide and analyzed by flow cytometry. The percentage of live cells was determined and the median lethal concentration (LC50) calculated (Fig. 2). Compared to wild type (WT) aerolysin, all of the modified versions showed a slight decrease in toxicity. The difference was greatest for AeL^C^, which was ∼10 fold less toxic than the WT. Modifying the N terminus with a single glycine impaired toxicity ∼3 fold. This was comparable to the loss of activity observed for the C-terminal modified version. Modification of both the N and C terminus of proaerolysin reduced toxic activity further and revealed the toxicity of ^N^proAeL^C^ to be intermediate between the WT and AeL^C^.

### Installation of a single label on proaerolysin

**Figure 3:**
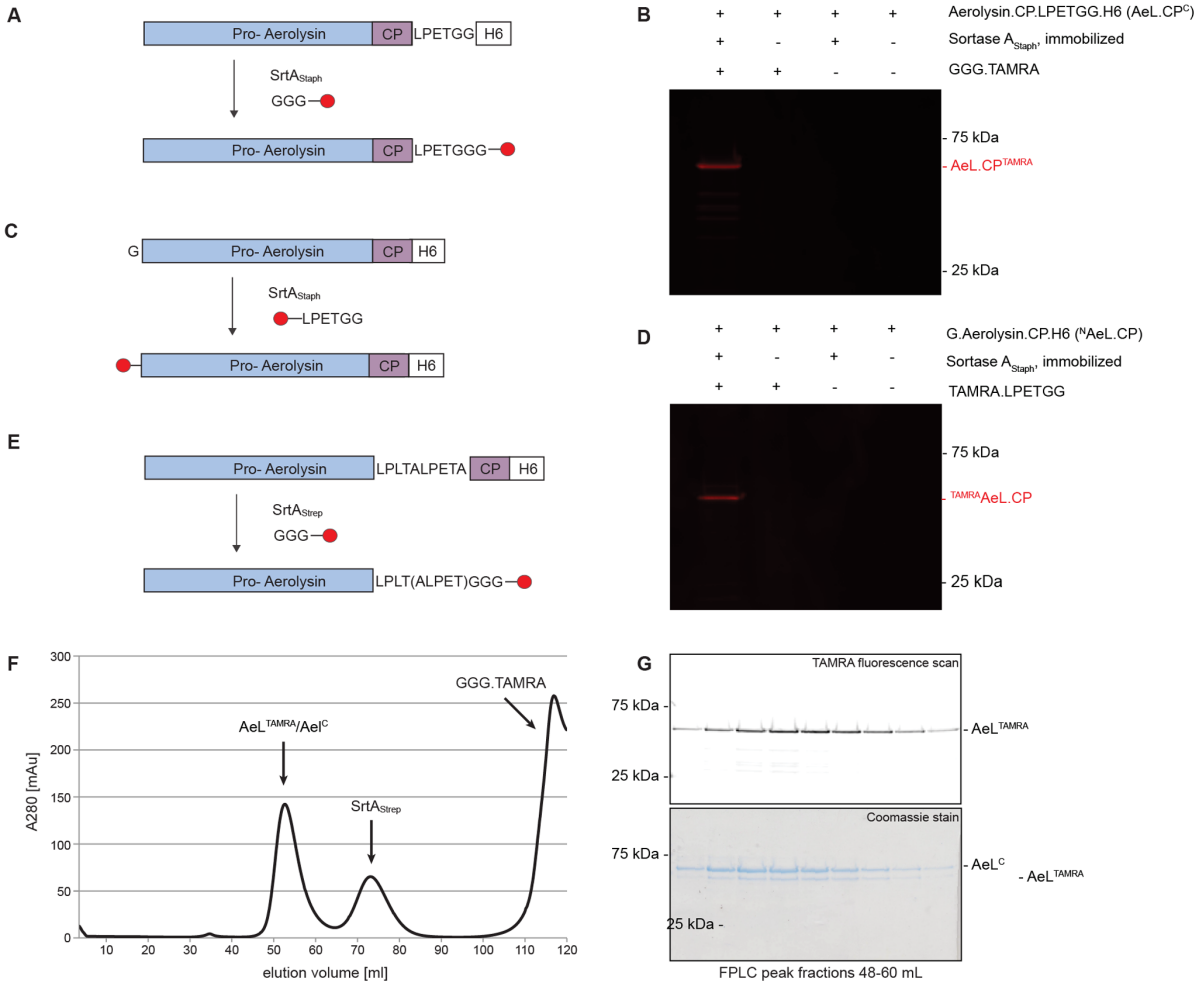
Installation of a single label on proaerolysin. The fluorophore carboxytetramethylrhodamine (TAMRA) was installed at the N-terminus of aerolysin (^N^AeL.CP), at the C-terminus of aerolysin upstream of the CP (AeL^C^) and at the C-terminus of the C-terminal peptide (AeL.CP^C^) with sortase. **A, C, E** Schematic representation of the sortagging reactions using of ^N^AeL.CP, AeL.CP^C,^ AeL^C^ respectively. **B, D** Sortagging of ^N^AeL.CP and AeL.CP^C,^ respectively, with respective control conditions, resolved by SDS PAGE and imaged with a fluorescence scanner. Product is visible by fluorescent signal. SrtA_Strep_ and SrtA_Staph_ recognize and cleave LPXTA and LPXTG motives, respectively. **F** Purification of labeled AeL^TAMRA^, gel filtration. The first peak in the A280 elution profile corresponds to aerolysin, the second to sortase, and the third to free nucleophile. **G** Analysis of the first peak of the gel filtration elution profile with SDS PAGE followed by fluorescence image scan and Coomassie stain. A fraction of Ael^C^ is not converted to fluorescent product.

Proaerolysin was labeled at either its N- or C-terminus with a peptide coupled to carboxy-tetramethylrhodamine (TAMRA) [Figs. 3*A and 3C*]. Fluorescent product was observed only when all the components of the labeling reaction mixture were co-mixed. No background labeling detected (Fig. 3*B and 3D*). The labeling efficiency was near-quantitative, as previously demonstrated for cholera toxin [32] and various other proteins [30] [33]. N-terminally labeled proaerolysin (^Tamra^AeL.CP) migrated slightly faster on SDS-PAGE than the C-terminally labeled AeL.CP^TAMRA^.

To label AeL^C^, we introduced a tandem sortase recognition site, LPLTALPETA, upstream of the protease cleavage site(s) that precede(s) the C-terminal peptide (Fig. 3E). We empirically determined that installation of a single sortase recognition motif, either LPLTA or LPETG, was insufficient to yield a good substrate for sortase and failed to yield a labeled product (data not shown).

Sortagged product was purified by fast protein liquid chromatography (FPLC) to separate the product from free dye-conjugated nucleophile and sortase (Fig. 3*F*). The fractions of the elution profile containing aerolysin were resolved by reducing SDS-PAGE and analyzed by fluorescence scan followed by coomassie staining. For this construct, labeling was incomplete (yield <50%) (Fig. 3*G*). Prolonged incubation times, different reaction temperatures and increasing the concentration of nucleophile did not further improve the extent of labeling (data not shown).

### Double-labeling of proaerolysin

**Figure 4:**
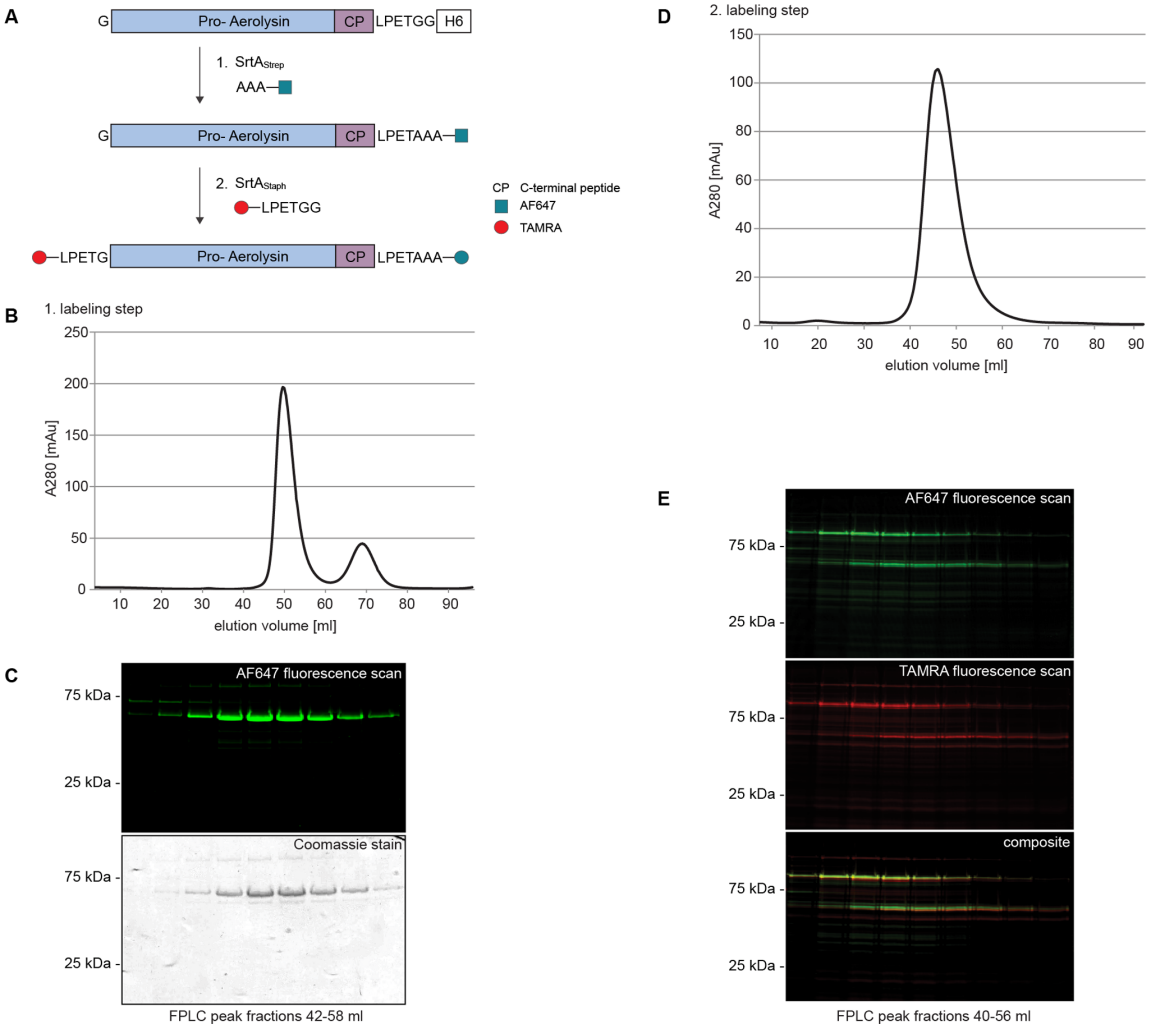
Double-labeling of proaerolysin. Double-labeling was achieved with a two-step approach. **A** Schematic representation of the dual labeling strategy of proaerolysin. **B** We used SrtA_Strep_ to install an oligoalanine coupled to the fluorophore AF647 at the C-terminus of proaerolysin, followed by a gel filtration purification step. **C** Elution profiles were analyzed by SDS-PAGE, fluorescence scan and coomassie stain. **D** The reaction product was subjected to the second round of sortagging with SrtA_Staph7M_ and LPETG-coupled TAMRA fluorophore for N-terminal labeling. SrtA_Staph_ does not recognize or cleave LPXTA, hence the C-terminal label remains intact. A single peak is observed on the elution profile as immobilized sortase was used for the reaction and removed prior to gel filtration. **E** Elution profiles were analyzed by SDS-PAGE followed by fluorescence scan.

Labeling with two different probes was achieved by combining sortases with different specificities, SrtA_Staph_ and SrtA_Strep_, such that the product of the first reaction was not recognized as a substrate for the second (Fig. 4*A*). In the first step, the C-terminus of ^N^AeL.CP^C^ was reacted with AAA.Alexa Fluor 647 by SrtA_Strep_ with near-complete labeling efficiency. The product was purified by FPLC and used as a substrate for the second labeling reaction (Fig. 4*B*). The elution peak containing ^N^AeL.CP^AF647^ also contained a minor fraction of higher and lower molecular weight species (Fig. 4*C*), the identity of which is not known.

TAMRA.LPETGG was appended to the N-terminus of ^N^AeL.CP^AF647^ in a second labeling step. We used immobilized SrtA_Staph7M_ to simplify sortase removal. Free nucleophile was removed by size exclusion chromatography (Fig. 4*D*). Labeling was monitored by SDS-PAGE, followed by fluorescence imaging (Fig. 4*E*). Two prominent polypeptides were visible in both channels (AF647: peudo color green; TAMRA: pseudo color red), one around 50 kDa, and a second around 100 kDa. In addition, a third polypeptide with an apparent molecular weight of 150 kDa was detected in the TAMRA channel but not in the AF647 channel. Image overlay showed co-localization of the 50 and 100 kDa species, most probably oligomers that lost the CP.

#### Aerolysin imaging

**Figure 5:**
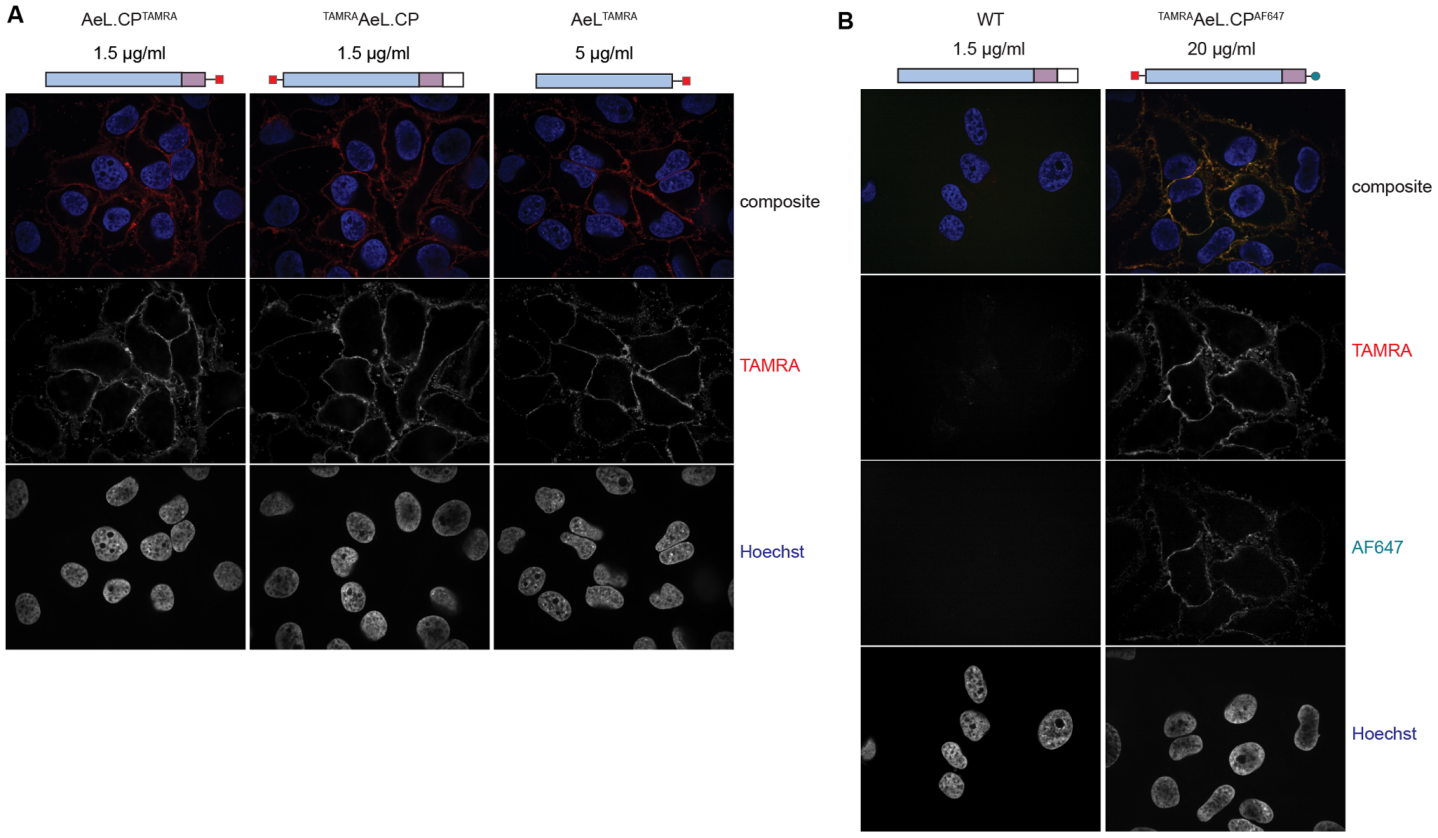
Aerolysin imaging. Aerolysin variants, fluorescently labeled, bind to the cell surface of HeLa cells. Images were acquired by confocal fluorescence microscopy. **A** Single labeled aerolysin versions**.** For comparable signal intensity, different aerolysin concentrations were required as indicated. **B** Double-labeled aerolysin and unlabeled aerolysin control.

Next we checked whether the different labeled proaerolysin versions would still bind to cells. Cell preparation, incubation, and the subsequent washing steps prior to fixation were done at 4°C to prevent activity of cell surface proteases that would otherwise activate proaerolysin. Confocal fluorescence microscopy revealed a rim-staining pattern for single-labeled proaerolysin (Fig. 5*A*). To acquire images with the same image acquisition settings (laser intensity, exposure time, gain), 3.3 times more (5 µg/mL) AeL^TAMRA^ had to be added to cells compared to both ^TAMRA^AeL.CP and AeL.CP^TAMRA^ (1.5 µg/mL). 20 µg/mL double-labeled ^TAMRA^AeL.CP^AF647^ was required for an adequate signal to noise ratio (Fig. 5*B*). Both fluorophores were visible as rim staining, and co-localized at the plasma membrane. Shifting the intoxicated cells to 37°C for 10 minutes prior to imaging resulted in cell detachment, indicative of intoxication (data not shown).

#### Dissociation of the C-terminal chaperone in the course of intoxication

**Figure 6:**
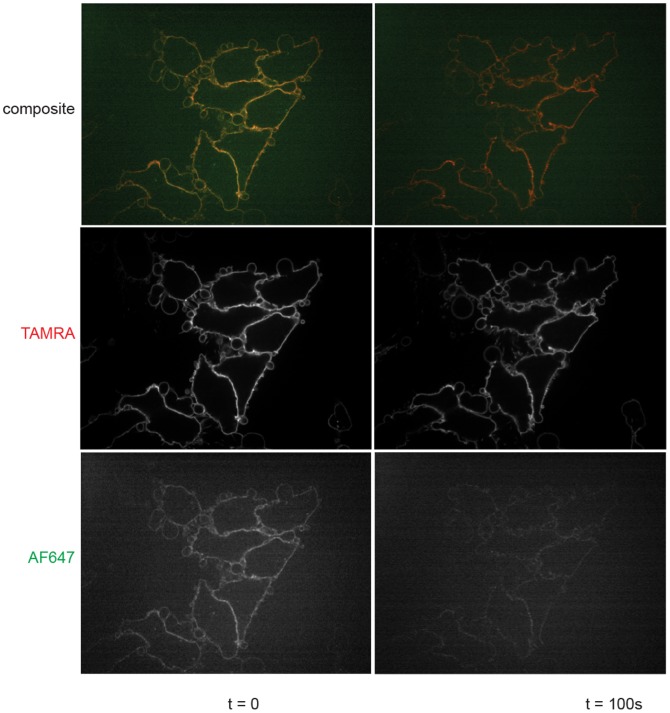
Dissociation of the C-terminal chaperone in the course of intoxication. HeLa cells were incubated with ^TAMRA^AeL.CP^AF647^ for 30 minutes at 4°C, washed, and the temperature shifted to 37°C. Images were acquired by confocal microscopy.

We used the double-labeled version of aerolysin to monitor the fate of the C-terminal chaperone during aerolysin intoxication. HeLa cells were incubated with ^TAMRA^AeL.CP^AF647^ for 30 minutes on ice, washed, and then shifted to 37°C. Confocal microscopy showed an initial overlapping surface staining pattern for both fluorophores. The intensity of the AF647 signal decreased over time to almost background level in ∼120 seconds, whereas the signal for TAMRA suffered loss of intensity to a much smaller extent and remained well above background (Fig. 6). This is indicative of separation of the two labels, and hence consistent with loss of the C-terminal peptide.

#### Identification of new aerolysin receptors

**Figure 7:**
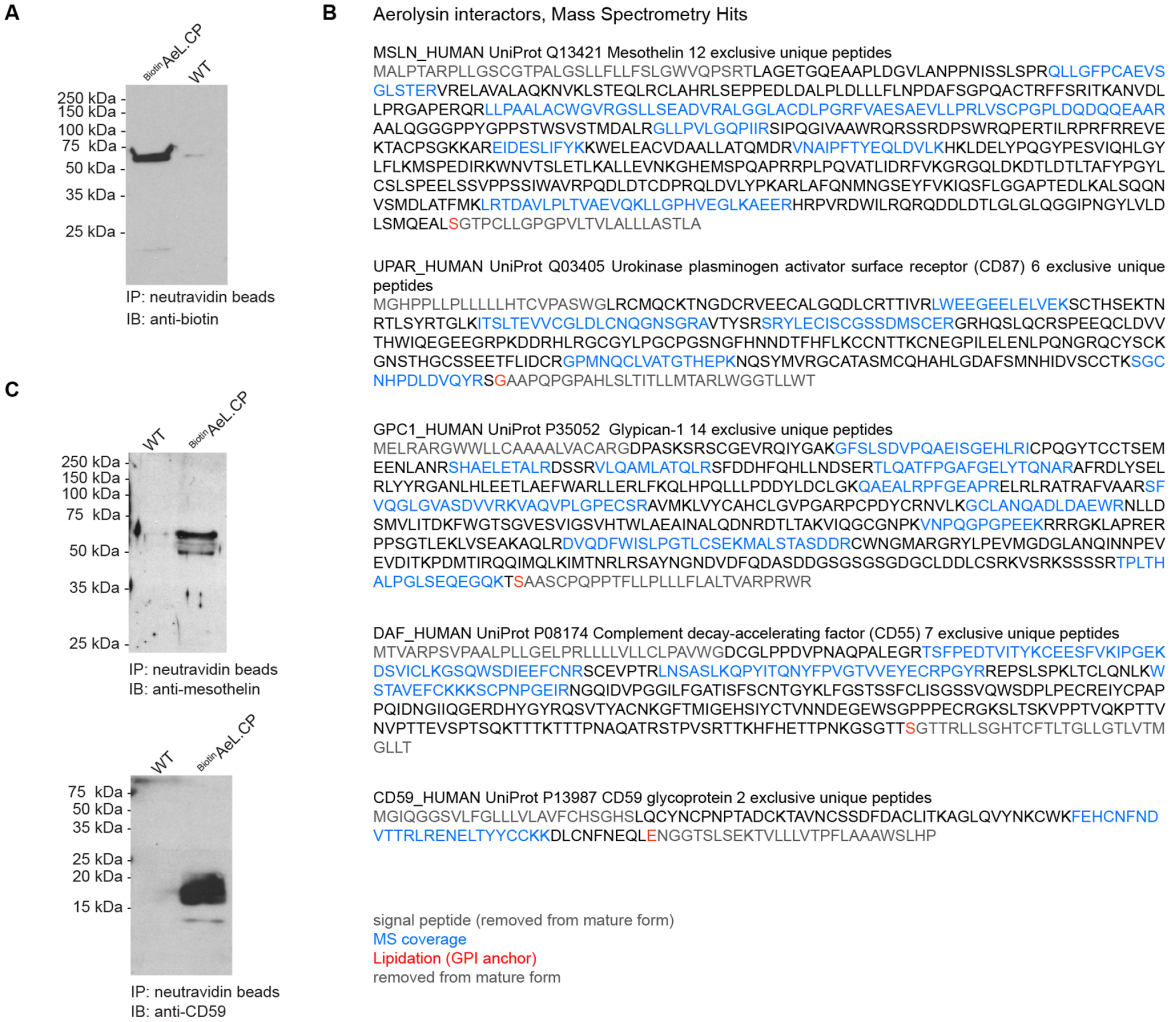
Identification of new aerolysin receptors. ^Biotin^AeL.CP was used to identify new GPI-anchored proteins that bind Aerolysin**. A** Biotin.LPETG was attached to the N-terminus of proaerolysin via sortagging. The purified reaction product was analyzed by immunoblot. **B** HeLa cells were incubated with ^Biotin^AeL.CP for 3 hours at 4°C and subsequently lysed with 0.5% NP-40. After pull-down with neutravidin beads, proteins were eluted, analyzed by SDS-PAGE, and subjected to mass spectrometry. Five GPI-anchored proteins were identified. UniProt accession codes are indicated. Peptides identified by mass spectrometry, lipidated amino acids, signal peptides, as well as peptides cleaved off from the pro-proteins are highlighted. **C** Binding of ^Biotin^AeL.CP to mesothelin and to CD59 was verified by immunoblot.

Aerolysin was sort°gged with biotin at its N-terminus (Fig. 7*A*) and incubated with HeLa cells at 4°C. Upon cell lysis, using a mild detergent, biotinylated aerolysin and its bound materials were recovered with neutravidin beads. The eluted proteins were separated on a reducing SDS-PAGE gel, and analyzed by mass spectrometry. Five GPI-anchored proteins were identified: mesothelin, urokinase plasminogen activator surface receptor (uPAR, CD87), glypican-1, complement decay accelerating factor (CD55), and CD59 glycoprotein; each represented by multiple exclusive unique peptide coverage (Fig. 7*B*). Interaction was confirmed for mesothelin and CD59 by immunoblot in an independent experiment (Fig. 7*C*).

## Discussion

Aerolysin is the first example of a pore-forming toxin to which a site-specific, chemoenzymatic labeling strategy has been applied. Sortagging allows maximal versatility in the choice of functionalities to be installed [28] [33]. Sortase accepts protein substrates in their native tertiary or quaternary structure. This eliminates two common problems of genetic fusion proteins: aggregation and non-functional folding. Aerolysin tolerates only subtle modifications [34]. A dramatic conformational change must take place for the soluble aerolysin monomer to form the homo-heptameric pore upon binding to a suitable receptor [17]; a point mutation can lock the protein into a particular conformation [35] and/or impede oligomerization [9], preventing toxicity. This leaves only three sites readily amenable to site-specific alteration: the very N-terminus, the very C-terminus, and the slightly more variable sequence that flanks the protease cleavage site(s) preceding the C-terminal peptide [36]. While it is true that addition of a few residues, a single glycine at the N-terminus of aerolysin, or the LPXTG/A motif diminishes toxicity (anywhere from a factor of 3 up to a factor of 10, Fig. 2), we have shown that enzymatic modification of any of these three aerolysin sites nonetheless yields a functional product fully capable of intoxication. It is not immediately obvious from the aerolysin crystal structure whether the N-terminus is critically engaged in receptor binding or pore-formation [37]. Genetic appendage of an affinity tag at the C-terminus of the C-terminal peptide for purification purposes is standard, but its effect on toxicity has not been systematically investigated. As observed in this study, internal modification of aerolysin has the most detrimental effect. The anomalous mobility on SDS-PAGE of ^TAMRA^proAeL compared to proAeL^TAMRA^ (calculated molecular weights: 55.5 kDa for ^TAMRA^proAeL and 54.8 kDa for AeL.CP^TAMRA^) we attribute to the relative positioning of the fluorophores, and the incomplete denaturation and/or differences in SDS binding. Alternatively ^TAMRA^AeL.CP may have lost its C-terminal peptide, which has a molecular weight of approximately 3.7 kDa. However, unless proteases were present in the sortagging reaction, or aerolysin could somehow activate autocatalytically, this we consider less likely. AeL.CP^TAMRA^ was clearly not affected in this manner, or it would have lost its fluorescence.

We show that modification of mature aerolysin not only at its N-terminus and C-terminal end of the CP (in the context of the holotoxin), but also at the newly generated C-terminus after cleavage of the CP is readily achievable using sortase. After the CP is cleaved off by the sortase reaction, it remains associated with non-receptor-bound aerolysin and continues to exert its function as a chaperone. By inhibiting aggregation and premature pore-formation, it maintains the molecule’s toxic potential [10].

Introduction of five additional amino acids in addition to the sortase recognition site was necessary to achieve successful sortase-mediated modification. Placing the LPXTG/A pentapeptide in a flexible loop generally increases flexibility of the protein backbone and thus accessibility, which in certain cases is required for proper sortase action [28] [32]. We did not determine which of the two motifs within this tandem sequence was recognized and used by sortase. The fact that modification of this site by addition of amino acids only modestly affects toxicity suggests that the new C-terminus has no critical function in the mature pore. Still, even though the five amino acid extension rendered the site accessible to sortase, the sortagging reaction could not be driven to completion. Presumably there is residual steric hindrance that interferes with accessibility for sortase.

Site-specific N- and C-terminal labeling of a single polypeptide using sortases of different specificity has been demonstrated in earlier work [29]. Applying the same strategy on aerolysin, obtained data are entirely consistent with double labeling. SrtA_Strep_ not only accepts oligoalanine, but also oligoglycine as a nucleophile, albeit with different kinetics [29] [38]. The N-terminal glycine of ^N^AeL.CP^label^ acts as a nucleophile, and can resolve the substrate-sortase thioacyl intermediate. This may result in concatenation of a fraction of aerolysin monomers, and would explain the detection of the 100 kDa and 150 kDa protein bands in the double-labeling reactions. These molecular weights are compatible with dimer and trimer formation, respectively. Dimers of aerolysin appear to contribute to the protein’s stability and have been detected in solution [39] [40] [41]. Dissociation in the presence of SDS is dependent on detergent concentration. Van der Goot et al. reported that “the dimer begins to come apart at 0.0125% SDS and is nearly completely dissociated by 0.025% detergent” [39]. The SDS concentration in our system is 0.1% (w/v), which should be sufficient to achieve denaturation. However, we know of several examples where non-covalent oligomers might be formed in the presence of SDS or resist to denaturing conditions, for example, the Cholera toxin B subunit [21]. How the enzymatic modification of aerolysin affects these properties is not known.

Installation of fluorescent tags does not compromise the ability of aerolysin to bind to its receptors. The different toxin amounts required to achieve equivalent binding reflect the differences in LC50 observed for the unlabeled, sequence-modified aerolysin variants. Moreover, the immediate detachment of the adherent HeLa cells shortly after temperature shift is a clear indication of the toxicity of the aerolysin variants. In the case of AeL^TAMRA^, where the unlabeled fraction constitutes the majority after reaction, it is not possible to infer toxicity of the labeled fraction, although the unlabeled, altered sequence of the input aerolysin preparation used for labeling is of course toxic.

With the double labeled ^TAMRA^AeL.CP^AF647^ construct in hand, we could not only confirm the toxicity of the labeled fraction itself, but also visualize the loss of aerolyin’s C-terminal peptide in the course of intoxication by microscopy. We thus confirm the previous findings of van der Goot et al that the chaperone is not part of the functional pore and separates from the active toxin [18].

A further application of sortagged aerolysin is the identification of new GPI-anchored human cell surface proteins that serve as receptors for the toxin. Previously, it was known that aerolysin binds to a subset of N-glycanated GPI-anchored proteins [11] [12] [42], where not only the GPI anchor, but also the receptor polypeptide moiety plays a role [13]. Plasma membrane microdomains act as a concentration platform for such GPI-anchored proteins [43]. The earliest identified receptor was Thy-1 from mouse lymphocytes [44]. Others are an unidentified 80 kD protein on baby hamster kidney cells [6], an unidentified 47 kD receptor on rat erythrocytes [45], the variant surface glycoprotein (VSG) of *Trypanosoma brucei* over-expressed in mammalian cells, *Leishmania major* CD63, but only when expressed in Chinese hamster ovary cells [11], and murine contactin [46]. In addition, aerolysin binds to human complement decay accelerating factor (CD55) [47]. In our assay we were able to detect CD55, attesting to the power of our approach, along with mesothelin, urokinase plasminogen activator surface receptor (uPAR, CD87), glypican-1, and CD59 glycoprotein, a novel set of molecularly identified GPI-anchored proteins not previously associated with aerolysin binding. Of note, CD59 was specifically excluded as an aerolysin receptor in previous work [13]. We speculate that the reason for this observed difference might be of a technical nature. The fact that we identify CD59 with two different analysis methods, immunoblot and mass spectrometry, makes us confident that CD59 is a true interaction partner of aerolysin.

Sortagging converts aerolysin into a versatile and valuable tool to study the ‘GPI-ome’ as a means of further characterizing lipid rafts where most GPI-anchored proteins are clustered [48], The sortagging strategy described here should be applicable also to other members of the bacterial pore-forming toxin family and may facilitate further biophysical studies on membrane interactions and pore formation.
